# Factors Affecting Postoperative Satisfaction After Presbyopia-Correcting Intraocular Lens

**DOI:** 10.3390/jcm15010336

**Published:** 2026-01-02

**Authors:** Choul Yong Park

**Affiliations:** Department of Ophthalmology, Samsung Medical Center, Sungkyunkwan University School of Medicine, Seoul 06351, Republic of Korea; oph0112@gmail.com

**Keywords:** presbyopia, intraocular lens, cataract, multifocal, satisfaction, EDOF

## Abstract

Presbyopia-correcting IOLs have revolutionized cataract surgery by enabling functional vision across multiple focal distances, thereby reducing dependence on spectacles. These lenses—ranging from multifocal to extended depth-of-focus (EDOF) and hybrid designs—incorporate advanced optical technologies to address the limitations of traditional monofocal IOLs. Despite their clinical promise, patient satisfaction remains variable, with a substantial subset experiencing postoperative visual discomfort. This review provides a comprehensive overview of presbyopia-correcting IOL technologies, detailing their optical principles, design evolution, and clinical performance. It further analyzes the multifactorial causes of postoperative dissatisfaction, which include optical phenomena such as glare, halos, and reduced contrast sensitivity; ocular comorbidities like dry eye disease, corneal irregular astigmatism, glaucoma, and macular pathology; and surgical variables including IOL centration, pupil size, and biometry accuracy. Additionally, non-physiological factors—such as patient expectations, lifestyle demands, and psychological disposition—play a critical role in perceived outcomes. To address these challenges, the review explores evidence-based strategies for improving satisfaction. These include rigorous preoperative screening for ocular surface disease and aberrations, personalized lens selection based on anatomical and functional criteria, and thorough patient counseling to align expectations with achievable results. Emerging IOL designs that blend multifocal and EDOF features offer promising avenues for minimizing visual disturbances while preserving range of vision. By integrating optical innovation with individualized clinical care, ophthalmologists can enhance postoperative outcomes and optimize real-world satisfaction with presbyopia-correcting IOLs.

## 1. Introduction

The global population is aging, and demand for presbyopia-correcting intraocular lenses (IOL) has grown accordingly [1]. Traditional monofocal intraocular lenses (IOLs) restore distance vision, whereas modern presbyopia-correcting IOLs aim to provide functional vision for near, intermediate, and distance tasks [2,3]. Advances in optical design, materials, and manufacturing have driven a transition from simple bifocal concepts to multifocal, extended depth-of-focus (EDOF), hybrid, and accommodating designs, meeting patients’ needs for spectacle independence in everyday activities such as reading, computer work, and smartphone use [2,3,4,5].

Presbyopia correction began with focal-splitting designs that created discrete distance and near foci [4]. Later generations added apodization, refined diffractive profiles, aspheric and chromatic aberration compensation, and toric options for astigmatism [4,5]. Recent innovations emphasize continuous or broadened focal ranges via wavefront engineering [6] and free-form diffractive surfaces, [7] and hybrid lenses that merge multifocality with EDOF features have emerged to balance near acuity with intermediate performance [8,9,10]. Improvements in biocompatible acrylics, edge design, and surface treatments have enhanced optical quality and mitigated some adverse effects [9].

Despite their capacity to improve independence and quality of life, presbyopia-correcting IOLs do not ensure universal satisfaction [11,12]. Optical trade-offs in multifocal and energy-splitting designs include reduced contrast sensitivity, glare, halos, and other photic phenomena that are particularly troublesome under low-light conditions [13,14]. EDOF solutions mitigate some of these effects but may provide less near acuity than traditional multifocal IOLs, introducing different compromises [3]. Objective acuity can be excellent while subjective visual quality and functional performance remain highly variable.

Multiple factors determine postoperative satisfaction [11,15,16,17]. Optical factors include preexisting corneal irregularities, higher-order aberrations, pupil dynamics, angle kappa, residual refractive error, and lens centration, tilt, or rotation [11,18]. Ocular comorbidities such as macular disease, glaucoma, or ocular surface disease can blunt the benefits of advanced optics [17]. Surgical factors including incision placement, biometry accuracy, and effective lens position are critical to achieving intended refractive outcomes [19]. Non-optical determinants—patient expectations, visual habits, occupational demands, personality traits, and mental health—strongly influence perceived success [11,19,20].

This review briefly summarizes the major categories of presbyopia-correcting IOLs and their optical principles, then examines the multifactorial contributors to postoperative dissatisfaction from optical, surgical, ocular, and psychosocial perspectives. The goal is to provide practical guidance for patient selection, counseling, and surgical planning to maximize real-world satisfaction with presbyopia-correcting lenses.

## 2. Presbyopia-Correcting IOLs

Recent advances in IOL technology have transformed cataract surgery from merely removing an opaque lens to actively correcting presbyopia. Unlike monofocal IOLs, presbyopia-correcting IOLs provide functional vision at distance, intermediate, and near, markedly improving postoperative convenience for patients [5]. However, their more complex optical designs can produce visual disturbances such as halos and glare and reduced contrast sensitivity, which are commonly encountered clinically.

Presbyopia-correcting IOLs have been continuously evolving. Early models’ drawbacks have been progressively addressed in newer designs, which aim to expand the functional range of vision while reducing patient-reported visual disturbances. Currently, the presbyopia-correcting IOLs widely used worldwide can be broadly divided into two main categories: multifocal (including bifocal and trifocal) designs and EDOF or hybrid designs [3,8,14].

Multifocal IOLs can be broadly categorized into refractive and diffractive types [4,5]. Refractive multifocal IOLs are often rotationally symmetric or asymmetric, with early designs typically providing near power in the inferior half of the optic and distance power in the superior half [21]. Zonal refractive type of multifocal IOL was also introduced [22] These bifocal configurations have since evolved into more advanced refractive trifocal models, offering improved visual performance across multiple distances [14]. Diffractive multifocal IOLs utilize the principle of light diffraction, manipulating the height and spacing of concentric diffractive rings to generate distinct focal points [4]. Initial designs provided far focus through the zero-order diffraction and near focus through the first-order diffraction, characteristic of bifocal IOLs [4]. Subsequent innovations have led to trifocal and even quadrifocal IOLs, achieved by combining multiple diffractive ring profiles to distribute light energy across several focal distances [14,23,24]. A fundamental consequence of multifocal optics is the simultaneous presentation of multiple focal points, which inherently increases the likelihood of dysphotopsia, such as halos and glare [2]. To mitigate these effects, advanced optic designs have incorporated apodization techniques—gradually varying the height of diffractive rings from the central optic zone (critical for near vision) to the peripheral zone (important for distance vision) [14]. Additionally, the sharpness of the kinoform structures that constitute each diffractive ring has been softened to reduce the intensity of photic phenomena [24,25]. These design refinements aim to balance multifocal functionality with improved visual comfort and reduced postoperative disturbances (Table 1).

EDOF IOLs create a single, continuous elongated focal region that extends depth of focus from distance to near, improving intermediate and near vision while largely preserving distance acuity [26]. Unlike monofocal IOLs, which focus light at one point, or multifocal IOLs, which produce two or three discrete foci with overlapping out-of-focus images that cause halos, EDOF designs avoid secondary out-of-focus images and reduce halo phenomena [26]. By distributing light energy along an extended focus rather than concentrating it at discrete foci, EDOF IOLs can slightly reduce contrast sensitivity and retinal image quality, representing a trade-off between depth of field and peak visual clarity [26]. EDOF IOLs fall into five types. Pure SA-based designs increase depth of focus by deliberately inducing positive or negative spherical aberration. Small-aperture lenses use a pinhole effect to extend acuity range. Low-addition multifocals are essentially multifocal IOLs with reduced near addition. Hybrid EDOF–MF lenses combine spherical aberration manipulation with a modest near add. Central-zone geometry lenses alter central versus peripheral refractive power or modulate the wavefront to elongate the focal region and enhance depth of focus [26] (Table 2).

Multifocal IOLs and EDOF IOLs both produce a visual experience distinct from the single-focus vision of a normal human eye. With multifocal IOLs, images for far, intermediate, and near foci are always presented simultaneously and neuroadaptation—where the brain learns to select the focal image it needs—is essential. Without successful neuroadaptation, patients will experience discomforting halos and other dysphotopsias from the simultaneous far, intermediate, and near images. The development of EDOF optics was driven in part by a desire to minimize multifocal IOL-related dysphotopsia by extending depth of focus through optical design to prioritize far and intermediate vision [3,26]. Even so, EDOF designs can sacrifice a portion of distance acuity—similar to the compromises seen with astigmatism between the circle of least confusion and the conoid of Sturm—and pure EDOF IOLs have struggled to achieve more than about 1 diopter of extended depth [3]. Recently, hybrid designs combining multifocal and EDOF technologies have been introduced and actively investigated to capitalize on the strengths of each approach while mitigating their respective limitations [4].

Presbyopia-correcting IOLs offer the advantage of providing patients with functional vision across multiple focal distances. However, compared to monofocal IOLs, they are associated with a higher incidence of dysphotopsia, including glare and halos, which may lead to increased dissatisfaction among patients who struggle to adapt to these visual phenomena [13,14]. Additionally, the risk of reduced visual acuity due to residual refractive errors, such as significant astigmatism, is also elevated with presbyopia-correcting IOLs.

Khandelwal et al. conducted a meta-analysis comparing visual disturbances between presbyopia-correcting and monofocal IOLs, revealing that presbyopia-correcting IOLs were associated with significantly worse outcomes for glare with a pooled risk ratio of 1.36 (95% CI: 1.15–1.61), and halos with a pooled risk ratio of 3.14 (95% CI: 1.63–6.08) [27]. This study incorporated data from multiple investigations utilizing a range of presbyopia-correcting IOLs, including Array SA40 (Johnson & Johnson, New Brunswick, US), Rezoom NXG1 (Johnson & Johnson, New Brunswick, US), Truevista (Storz, Tuttlingen, Germany), Isert PY60MV(Hoya, Tokyo, Japan), 815LE (3M), 808X (Pharmacia, Uppsala, Sweden), Progress 3 (Laboratories Domilens, Lyon, France), Tecnis ZM900 (Johnson & Johnson, New Brunswick, US), ResSTOR SA60D3/SN6AD1/SN6AD3 (Alcon, Fort Worth, US), Acri-Lisa 366D (Zeiss, Oberkochen, Germany), and Acri.tec Twin (Zeiss, Oberkochen, Germany). Ukai et al. compared the photic phenomena between PanOptix (Alcon, Fort Worth, US) and Synergy (Johnson & Johnson) implanted patients [16]. The study revealed that while glare levels were comparable between the two groups, the Synergy group experienced halos that were larger and thicker, along with more pronounced and intense starbursts. In the PanOptix group, postoperative halo brightness showed a positive correlation with corneal coma aberration. Meanwhile, in the Synergy group, both the size and brightness of postoperative halos and starbursts were positively associated with pupil diameter.

While individual IOL designs exhibit distinct optical characteristics, general trends can be observed across categories. Multifocal IOLs are known to provide superior near visual acuity due to their discrete focal zones; however, they are also associated with a higher incidence of dysphotopsia—such as glare, halos, and starbursts—and typically require a longer period of neural adaptation [12,14]. In contrast, EDOF IOLs offer a more favorable profile in terms of minimizing dysphotopsia and facilitating smoother neural adaptation, owing to their continuous focal range and wavefront-optimized designs [26] Nevertheless, EDOF lenses tend to deliver comparatively weaker near vision performance, which may limit their effectiveness for patients with high near-vision demands.

The near-focus power of an IOL is influenced by its effective lens position (ELP). As the ELP increases, the functional add power of the IOL diminishes [28]. In eyes with longer axial length and steep keratometry, the ELP is typically located further posterior to the cornea compared with eyes that are emmetropic or hyperopic, thereby reducing the effectiveness of the add power [29]. Clinically, surgeons often compensate for this by setting the postoperative refractive target to approximately −0.50 to −0.75 D in highly myopic patients, which helps to enhance near vision [30]. Keratometry and axial length measurements allow estimation of the actual near focus distance in advance, aided by online software tool (https://sheet.zohopublic.com/sheet/published/r811sc159e83f7e6044e79f7ea7dacaa484d9 accessed on 17 December 2025). Careful evaluation of the patient’s near vision requirements before surgery is essential. If a long ELP is anticipated and the patient has strong demands for near vision, it should be explained that a multifocal IOL may provide superior near vision compared with an EDOF IOL, and the choice of lens should be made accordingly.

## 3. Ocular Surface Disease

### 3.1. Dry Eye Disease

Dry eye disease is frequently encountered among older individuals scheduled for cataract surgery. The Prospective Health Assessment of Cataract Patients Ocular Surface Study investigated the incidence and severity of dry eye disease (DED) in 136 patients (272 eyes) scheduled for cataract surgery. The findings revealed a notably high prevalence of DED, with 80.9% of participants exhibiting a severity grade of level 2 or higher according to the International Task Force Dry Eye Classification [31]. DED is not confined to the preoperative phase, as it can emerge de novo or exacerbate in the postoperative period following cataract surgery. Almost 30% of patients had persistent DED at 3 months postoperatively [32]. Villani et al. developed the Ocular Surface Frailty Index (OSFI, range 0.0 to 1.0) and assessed its predictive value for DED symptom onset after cataract surgery [33]. The study included 284 eyes of 284 patients without preoperative DED symptoms who underwent uneventful cataract surgery. OSFI results of 0.3 or more (but not age, gender, or any preoperative sign) was a good predictor of ocular surface symptom onset (odds ratio, 9.45). The Corneal Clinical Committee of the American Society of Cataract and Refractive Surgery (ASCRS) formulated a consensus-driven, practical diagnostic algorithm for ocular surface disease (OSD) to support surgeons in the effective identification and management of clinically significant OSD prior to performing refractive surgery [34].

DED-related tear film dysfunction may compromise visual quality and contribute to ocular discomfort, potentially leading to postoperative outcomes that fall short of patient expectations. It has been reported that 15% of patients who were dissatisfied after multifocal IOL implantation were found to have DED [17].

Postoperative management of DED has been shown to enhance both visual outcomes and overall patient satisfaction, according to recent clinical studies. Teshigawara et al. reported that perioperative management of DED with rebamipide eyedrop in patients undergoing cataract surgery with trifocal IOL implantation demonstrated significantly greater efficacy than artificial tears in enhancing postoperative ocular surface integrity, contrast sensitivity, and reducing disability glare [35]. Preoperative DED treatment combining manual meibomian gland expression with intense pulsed light improved tear film stability and patient satisfaction [36].

### 3.2. Cornea Irregular Astigmatism

Corneal topography identifies regular and irregular astigmatism, including higher-order aberrations (HOAs) like spherical aberration, as well as corneal shape abnormalities. Irregular corneal astigmatism is a contraindication for multifocal IOLs [37]. Greater HOAs were associated with poorer visual performance in low luminance and reduced contrast conditions [38]. It commonly results from prior corneal infections, trauma, dystrophies, pterygium, or severe DED [39]. In such cases, aberrometry analysis of cornea often reveals elevated HOAs over a central 6 mm zone [39]. In the previous study, the total HOA root mean square (RMS) in 770 normal eyes (mean age of 50.5 years) was reported as 0.484 ± 0.173 µm in 6 mm zone [40]. In another study analyzing 976 eyes from 976 patients (mean age of 65 years), the mean RMS of total HOAs measured at the central 4 mm and 6 mm optical zones were 0.20 μm and 0.65 μm, respectively [41]. If this value of HOAs RMS exceeds 0.50 μm in 6 mm zone, the risk of postoperative halos and glare with multifocal IOLs significantly increased [42]. Goto et al. set a tentative cutoff for multifocal IOL candidacy at corneal HOAs < 0.3 µm over a 4 mm pupil, since a 0.5 D defocus theoretically corresponds to an RMS wavefront error of approximately 0.29 µm for a 4 mm diameter [37]. In cases of significant HOAs, surgeons should counsel patients on the potential impact on postoperative visual outcomes. Multifocal IOLs are not recommended for such eyes. Using the Pentacam (Oculus, Wetzlar, Germany), Maeda et al. set the cut-off value in total HOAs for 4 mm diameter to 0.3 μm for mild irregular astigmatism, 0.5 μm for moderate irregular astigmatism [43].

EDOF optics are generally expected to tolerate small irregular corneas better than multifocal IOLs because of their depth-of-focus design. However, no direct comparative studies of clinical outcomes or patient satisfaction between EDOF IOL and multifocal IOLs in eyes with irregular corneal astigmatism have been reported to date. While EDOF IOL employing pinhole technology are theoretically anticipated to exhibit better tolerance to irregular astigmatism, published clinical studies validating this assumption are unavailable.

Alongside irregular astigmatism, another important corneal shape factor is spherical aberration. As described earlier, some EDOF IOLs utilize spherical aberration to enhance near vision. By mathematical approximation, 0.25 µm of negative spherical aberration with a 6 mm pupil diameter can result in at least 0.75 diopters of myopic defocus and 0.4 µm of negative spherical aberration can induce 2.0 diopters of myopic defocus, thereby enhancing reading ability [44]. The spherical aberration of a normal cornea has been reported in previous studies to range between 0.27 and 0.33 µm [45]. EDOF IOLs that utilize negative spherical aberration are designed based on the typical range of corneal spherical aberration observed in normal eyes. If a patient’s corneal spherical aberration deviates significantly from this norm, the intended extended depth of focus effect may not be achieved, potentially leading to suboptimal visual outcomes and increased patient discomfort.

## 4. Residual Refractive Errors

Residual refractive error following cataract surgery may arise from multiple factors, including inaccurate biometry techniques, erroneous estimation of effective lens position or axial length, surgically induced astigmatism, and IOL tilt or decentration. In cases where residual refractive error remains after surgery, the optimal performance of presbyopia-correcting IOLs may be compromised, often leading to increased patient dissatisfaction. Particularly, when significant refractive errors persist—such as a spherical or astigmatic error of ≥0.75 D—additional surgical intervention may be required to achieve satisfactory visual outcomes [46,47].

Schallhorn et al. compared eyes with 38,828 multifocal and 11,571 monofocal IOLs [48]. Residual refractive errors (≤−0.25 D myopia, ≥+0.50 D hyperopia) had a significant impact on uncorrected distance visual acuity across both monofocal and multifocal IOLs. Monofocal IOLs demonstrated a consistent improvement in uncorrected near visual acuity with each additional 0.25 D of myopia, whereas multifocal IOLs did not show a statistically significant change. The mean gain in near vision between 0.00 D and −1.00 D sphere was 0.26 logMAR for monofocal IOLs, compared to just 0.03 logMAR for multifocal IOLs. Low near-addition IOLs showed a modest improvement in near vision with increasing myopia, though less pronounced than with monofocal lenses. Notably, patient dissatisfaction associated with residual refractive error was more prominent in the multifocal IOL group.

EDOF IOL seems to be more tolerable to residual refractive errors [49,50]. Rementería-Capelo et al. studied 30 eyes with Acrysof^®^ IQ Vivity^®^ (Alcon, Fort Worth, US) implantation [49]. The study evaluated 30 eyes from 30 patients. Mean uncorrected and corrected distance visual acuities were −0.04  ±  0.05 and −0.05  ±  0.05 logMAR, respectively. Visual acuity with +0.50 D and −0.50 D defocus was 0.01  ±  0.06 and 0.00  ±  0.04 logMAR, respectively. Distance-corrected visual acuity was significantly better, with no significant difference between myopic and hyperopic defocus. Under astigmatic conditions, distance visual acuity was 0.01  ±  0.05 logMAR for against-the-rule, 0.01  ±  0.06 for oblique, and 0.01  ±  0.04 for with-the-rule astigmatism [49]. In a laboratory simulation applying 0.50 D of myopia, 0.50 D of hyperopia, and 0.75 D of astigmatism, the PureSee IOL demonstrated visual performance comparable to that of the TECNIS monofocal IOL, and superior to the TECNIS multifocal IOL [50].

## 5. Other Ocular Pathology

### 5.1. Glaucoma

Patients with glaucoma typically exhibit reduced contrast sensitivity and impaired mesopic visual function [51]. Since multifocal IOLs can also diminish these visual parameters, their implantation may lead to significant visual disturbances in this population [52]. Consequently, established glaucoma is generally regarded as a contraindication for multifocal IOL use. Patients implanted with diffractive multifocal IOLs exhibit a clinically significant reduction in visual sensitivity, as measured by standard automated perimetry. Sánchez-Sánchez et al. reported high spectacle dependence and low contrast sensitivity in patients implanted with multifocal IOLs compared to monofocal IOLs [53]. Such sensitivity loss can complicate the accurate evaluation of glaucoma progression, potentially affecting disease monitoring and management [54].

In mild or moderate glaucoma patients, EDOF IOL may provide better visual outcome compared to multifocal IOLs [55]. Urcola et al. analyzed visual outcomes of EDOF IOL (IQ Vivity, Alcon, Fort Worth, US) in patients with mild primary open-angle glaucoma [56]. Although binocular contrast sensitivity was found to decline more noticeably at high spatial frequencies than at low ones, 88.89% of patients reported “never” or “rarely” needing glasses for distance vision, 91.67% for intermediate tasks, and 63.89% for near vision [56]. Ferguson et al. implanted EDOF IOL (IQ Vivity, Alcon, Fort Worth, US) in patients with mild open-angle glaucoma [57]. They found that 92% of patients (*n* = 24) reported never needing glasses for daytime driving, and the same percentage applied to those who drove at night. For intermediate tasks such as computer use, 24% (*n* = 6) indicated frequent or consistent reliance on glasses, while 50% (*n* = 13) reported never using them. Regarding mobile phone use, responses were evenly split: 38% (*n* = 10) never required glasses, whereas another 38% (*n* = 10) frequently or consistently did [57]. Bissen-Miyajima et al. evaluated the performance of diffractive EDOF IOL (Symfony ZXROOV, Johnson & Johnson) in patients with normal tension glaucoma [58]. The study involved 16 eyes from 10 patients who were well-controlled on no more than two glaucoma medications, exhibited no central visual field defects, and had an average mean deviation of −4.78 dB (range: −0.79 to −12.25 dB) on Humphrey visual field testing. Their findings showed that EDOF IOL implantation provided distance visual acuity and contrast sensitivity outcomes comparable to those in non-glaucomatous eyes implanted with the same IOL [58].

The reduction in contrast sensitivity caused by presbyopia-correcting IOLs may interfere with the accurate assessment of visual field changes in patients with glaucoma. It is of note that implantation of Eyhance (enhanced monofocal IOLs, Johnson & Johnson) in patients with glaucoma did not lead to any noticeable reduction in visual sensitivity when compared to standard monofocal IOLs [55,59,60,61].

Previous studies indicated that presbyopia-correcting IOLs, compared to monofocal IOLs, tend to reduce contrast sensitivity and increase postoperative dependence on spectacles in patients with glaucoma. However, for individuals with preperimetric or mild glaucoma who strongly desire presbyopia correction, EDOF IOLs may be relatively tolerable. Nonetheless, these patients are more likely to experience visual discomfort after implantation compared to those without glaucoma, making thorough preoperative counseling essential.

### 5.2. Macular Degeneration and Epiretinal Membrane

Macular pathologies such as age-related macular degeneration (AMD) and epiretinal membrane (ERM0) are relative contraindications for multifocal IOL [42]. These patients are known to have an increased risk of reduced visual acuity, diminished contrast sensitivity, and discomfort caused by photic phenomena. Patel et al. implanted AcrySof IQ ReSTOR^®^ in 6 eyes with ERM and found visual recovery is delayed and two of them still complained metamorphopsia [62].

However, EDOF IOL can be used in selective cases with mild macular pathology. Compared to multifocal IOLs, several reports suggest that EDOF IOLs yield relatively more tolerable outcomes in patients with limited macular abnormalities [63]. Jeon et al. reported that eyes with low-grade ERM showed comparable outcomes to eyes without ERM after AcrySof^®^ IQ Vivity implantation [64]. Sararols et al. compared 22 eyes without ERM with 22 eyes with stage 2 or 3 ERM in the same patients after implanting AcrySof^®^ IQ Vivity. This bilateral comparison found there were no differences observed in visual outcomes, contrast sensitivity, or visual disturbances between ERM and non-ERM eyes [65].

Reitblat et al. implanted Tecnis Symfony in eyes diagnosed with mild ERM or mild dry AMD, classified as category 1 or 2 according to the Age-Related Eye Disease Study (AREDS) [66]. Their findings indicated that uncorrected visual acuity was significantly superior in eyes without macular pathology compared to those with retinal abnormalities. Spectacle independence was notably higher in the healthy cohort (71% vs. 38%), whereas the incidence of photic phenomena such as haloes and glare was more prevalent among eyes with macular pathology (23% vs. 17%). In contrast, Chung et al. reported that following the implantation of Tecnis Symfony intraocular lenses in 16 eyes with stage 1 or 2 ERM, none of the patients experienced visual disturbances indicative of photic phenomena, such as glare or halos [67].

When selecting IOLs for patients with macular pathology, it is essential to thoroughly explain that both multifocal IOL and EDOF IOLs may yield suboptimal outcomes due to their optical characteristics. Patients should be informed that visual improvement may be delayed compared to those without retinal abnormalities, and that final visual acuity may also be lower. Depending on the severity of the macular pathology, symptoms such as metamorphopsia or scotomas may remain after the surgery, and the associated visual discomfort could be more pronounced with multifocal IOLs and EDOF IOLs than with monofocal IOLs.

### 5.3. Cornea Endothelial Diseases

Inaccurate biometry due to corneal edema can lead to errors in determining the appropriate IOL power. This is particularly important in patients with Fuchs endothelial corneal dystrophy (FECD) or pseudophakic bullous keratopathy (PBK), where future corneal transplantation may reduce corneal edema and alter refractive outcomes. Such changes must be carefully considered when selecting the power of presbyopia-correcting IOLs. Additionally, corneal edema can increase light scatter and induce irregular astigmatism, potentially leading to patient dissatisfaction following presbyopia-correcting IOL implantation [68].

When planning cataract surgery in patients with corneal endothelial disease, effective patient counselling is critical to achieving favorable surgical outcomes, particularly in complex cases involving presbyopia-correcting intraocular lenses [69]. Adequate time should be allocated to ensure that patients fully understand the postoperative course and potential contingencies. They should be informed that recovery may take longer than typical cataract procedures, and that additional interventions—such as endothelial keratoplasty—may be necessary in the event of corneal decompensation. Furthermore, patients must be made aware of the importance of adhering to a schedule of regular follow-up visits to monitor healing, assess visual function, and promptly address any emerging complications [70].

Previous studies have demonstrated that the resolution of preoperative corneal edema following Descemet Membrane Endothelial Keratoplasty (DMEK) primarily influences the posterior corneal surface [71]. This is reflected by a forward shift in elevation maps and a central reduction in corneal thickness CT, while the anterior corneal curvature remains largely stable and unchanged from preoperative measurements [71,72]. Corneal refractive changes are more complex following Descemet stripping automated endothelial keratoplasty (DSAEK). The procedure introduces a donor lenticule of variable thickness to the posterior corneal surface, imparting optical properties that remain only partially understood during preoperative planning and IOL selection [73]. When combining cataract surgery and endothelial keratoplasty such as DSAEK or DMEK, it is recommended to set the postoperative refractive target slightly myopic (−1.0~−1.5 D target for DSAEK and −0.5~−1.0 D target for DMEK) [74,75,76]. Price et al. reported favorable visual outcomes when cataract surgery and presbyopia-correcting IOL implantation, using presbyopia-correcting IOLs (14 Symfony or and 2 ReSTOR+4), were staged after the DMEK [77]. However, another study reported that FECD patients implanted with EDOF IOLs showed worse uncorrected distance VA and better uncorrected near VA than controls, with lower spectacle independence for intermediate and overall ranges but no increase in photic phenomena [78]. In the multifocal IOL subgroup, both FECD and control groups demonstrated comparable visual acuity across all distances and achieved similar levels of spectacle independence. However, FECD patients reported a higher incidence of photic phenomena, which, importantly, did not interfere with their daily activities [78].

As mentioned, DSAEK introduces additional refractive complexity owing to the lamellar graft’s variable thickness and curvature, which are difficult to quantify accurately before surgery. As a result, the selection of presbyopia-correcting IOLs in these patients demands heightened caution and individualized evaluation. For patients with a strong desire for spectacle independence, EDOF IOLs may represent a preferable option, given their lower incidence of dysphotopsia and greater tolerance of optical imperfections compared with multifocal designs.

## 6. Pupil

Pupil diameter and postoperative IOL centration relative to the pupil significantly influence vision and patient satisfaction in presbyopia-correcting IOL surgery. The impact of pupil size and decentration differs among presbyopia-correcting IOLs owing to variations in optic design.

Fernández et al. evaluated photopic and mesopic pupil diameters in 168 eyes following multifocal IOL implantation [79]. Their findings revealed that 84.5% and 95.8% of eyes exhibited photopic pupil sizes of ≤3.0 mm and ≤3.5 mm, respectively. Under mesopic conditions, pupil sizes exceeded 4.5 mm in 39.3% of eyes and 5.0 mm in 16.7%. Additionally, both mesopic and photopic pupil diameters showed an age-related decline, decreasing by 0.28 mm and 0.15 mm per decade, respectively. In addition, at one month following cataract surgery, pupil size decreased, with photopic and mesopic pupils showing reductions of 9.8% and 9.1%, respectively [80]. Additionally, temporal centroid shift was reduced, with the mean value decreasing from 0.12 mm preoperatively to 0.05 mm postoperatively [80].

Tandogan et al. compared three types of IOLs using an optical bench setup: an aspheric monofocal IOL (CT ASPHINA 409M), an aspheric diffractive bifocal IOL (AT LISA 809M), and an aspheric diffractive trifocal IOL (AT LISA 839M) [81]. At far focus, the monofocal IOL showed the highest modulation transfer function (MTF) values of 0.80 for both 3.0 mm and 4.5 mm pupil apertures. In contrast, the bifocal and trifocal IOLs demonstrated lower MTF values at far focus—0.46/0.41 and 0.39/0.26, respectively—with a tendency for mild reduced performance as pupil size increased. However, at near focus, the bifocal IOL showed MTF values of 0.27/0.31 and the trifocal IOL 0.19/0.18, indicating relatively consistent optical performance regardless of pupil size.

Multifocal IOLs that utilize diffractive technology feature unique kinoform designs, which vary by manufacturer. These specialized designs significantly influence the optical performance at far, intermediate, and near distances. For example, the PanOptix TFNT00 (Alcon) distributes light energy at 58% for far, 19% for intermediate, and 22% for near vision, consistently across both 3.0 mm and 4.5 mm apertures [82]. In contrast, the Synergy (Johnson & Johnson) shows a different energy distribution: 43%/20%/37% (far/intermediate/near) for a 3.0 mm aperture and 43%/21%/36% for a 4.5 mm aperture [82].

**Figure 1:**
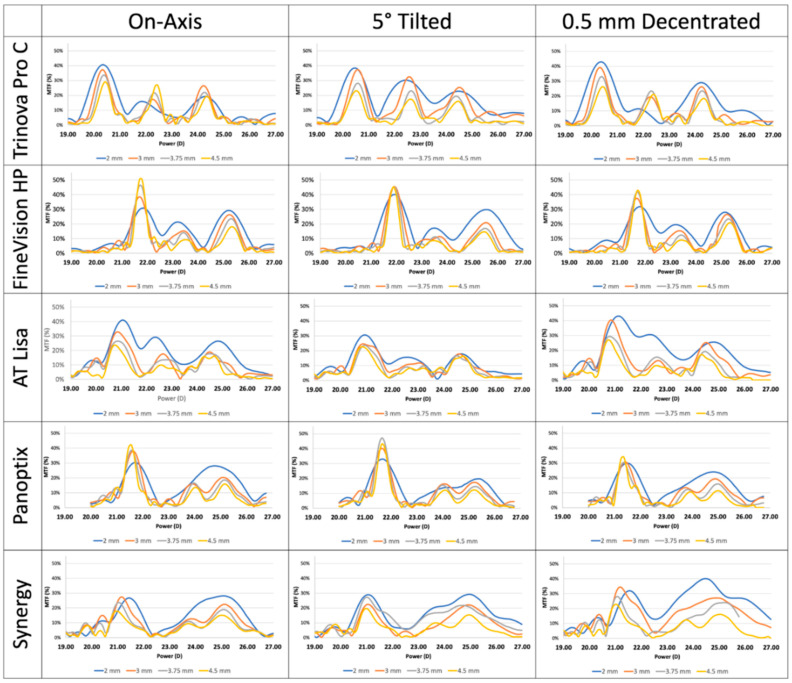
Through focus modulation transfer function (MTF) of five types of multifocal IOLs (Acriva Trinova (VSY-Biotechnology), FineVision HP (PhysIOL), AT LISA tri 839 MP (Zeiss), PanOptix TFNT00 IOL (Alcon), and Tecnis Synergy (Johnson & Johnson) according to simulation of different pupil sizes (2~4.5 mm), 5 degree tilt and 0.5 mm decentration. (Can et al. *Sci. Rep.* **2023**, *13*, 19646), copied under Creative Commons license) [82].

Can et al. compared the optical performance of five types of multifocal IOLs (Acriva Trinova (VSY-Biotechnology), FineVision HP (PhysIOL), AT LISA tri 839 MP (Zeiss), PanOptix TFNT00 IOL (Alcon), and Tecnis Synergy (Johnson & Johnson)) [82] (Figure 1). On-axis optical bench testing revealed that most IOLs maintained a through-focus modulation transfer function (MTF) above 0.3 at a 3 mm aperture, with the exception of the Tecnis Synergy lens. The FineVision HP and PanOptix demonstrated improved far-focus MTFs at larger apertures (3.75 mm and 4.5 mm), whereas the AT LISA Tri 839 MP provided superior intermediate and near-focus performance under the same conditions. When a 5° tilt was introduced, the Acriva Trinova retained better overall optical quality across all focal distances, particularly with medium-sized pupils (3 mm). In contrast, the AT LISA Tri and PanOptix lenses showed marked declines in intermediate-focus MTF under tilt conditions. Additionally, a 0.5 mm decentration significantly impacted the intermediate-focus performance of AT LISA Tri and Tecnis Synergy lenses, while FineVision HP and PanOptix lenses experienced notable degradation in far-focus MTF (Figure 1).

Baur et al. compared four different EDOF IOLs using an optical metrology instrument (OptiSpheric IOL PRO2; Trioptics GmbH) and found pupil dependency was more pronounced with the MiniWell, LuxSmart, and AcrySof IQ Vivity, whereas Lentis Comfort showed a more consistent behavior at different apertures [83].

One important consideration is that the center of the pupil does not always coincide with the center of the capsular bag. In clinical practice, precise localization of the center of the capsular bag is virtually impossible. Additionally, the pupil center is typically positioned temporally relative to the corneal apex, and under mesopic conditions, it tends to shift even further temporally compared to its photopic location [84]. However, IOL is inherently centered in the capsular bag due to its structural design. Studies have also reported that following cataract surgery, the pupil size decreased by approximately 9–10%, and the temporal shift of the pupil center (centroid shift) was reduced compared to the preoperative state [80]. This discrepancy between the pupil center and the IOL center may lead to visual discomfort in some patients. Particularly, certain presbyopia-correcting IOLs—owing to their specific optical designs—can be more sensitive to such misalignment.

Inadequate pupil size can adversely impact visual acuity following multifocal IOL implantation, as pupil diameter influences which optical zones of the IOL are engaged. Patients presenting with excessively small postoperative pupils and reporting poor near vision may benefit from treatment with cyclopentolate eye drops or a 360° argon laser iridoplasty. Ouchi et al. conducted a comparative analysis of contrast sensitivity following implantation of the TECNIS^®^ ZMB00, stratifying patients based on preoperative photopic pupil diameter [85]. The small-diameter pupil group included eyes with a pupil diameter ≤ 3.0 mm, while the large-diameter pupil group comprised those with diameters > 3.0 mm. The small-diameter pupil group demonstrated significantly reduced contrast sensitivity at high spatial frequencies (12 and 18 cycles per degree), as well as poorer low-contrast visual acuity at both 12.5% and 6% contrast levels, compared to the large-diameter pupil group.

Conversely, individuals with abnormally large postoperative pupils experiencing heightened photopic phenomena may be effectively managed with brimonidine tartrate 0.2% ophthalmic solution [14].

## 7. Tilt and Decentration of Multifocal IOL

Most trifocal IOLs introduce asphericity in one of the surfaces of the lens in order to induce some level of negative spherical aberration that might enhance the image quality by neutralizing the positive spherical aberration normally caused by the cornea [23]. However, this can make the IOLs less tolerant to tilts or decentration [86]. In addition, multifocal IOLs are more susceptible to the influence of tilt and decentration than monofocal IOLs due to the complexity of optical design. Refractive multifocal IOLs may be more affected by pupil size dynamics and decentration compared to diffractive multifocal IOLs [5].

Mild degrees of IOL tilt (2°–3°) and decentration (0.2–0.3 mm) are frequently observed in clinical studies and typically go unnoticed by most patients, including those with multifocal IOLs [87]. Baumeister et al. reported, using Scheimpflug photography, that implanted aspheric IOLs exhibited a mean optic tilt of 2.85° ± 1.36 and a mean decentration of 0.27 ± 0.16 mm [87]. For diffractive bifocal and trifocal lenses, optical quality at all distances was significantly reduced if decentration exceeded 0.75 mm [81,88].

Tandogan et al. [81] found MTF values for the monofocal IOL (CT ASPHINA 409M, Zeiss); far-focus MTF values were 0.80 at both 3.0 mm and 4.5 mm apertures and decreased to 0.77 and 0.73, respectively, when decentered by 1 mm. In comparison, far-focus MTF values for the bifocal IOL (AT LISA 809M, Zeiss) decreased from 0.46/0.41 at 3.0 mm and 4.5 mm apertures to 0.35/0.25 with 1 mm decentration. And far-focus MTF values for the trifocal IOLs (AT LISA 839M, Zeiss) decreased from 0.39/0.26 at 3.0 mm to 0.25/0.18 with 1 mm decentration [81].

EDOF IOLs are generally more resistant to the adverse optical effects of decentration and tilt than multifocal IOLs. Bellucci et al. found that for the MiniWELL Ready (SIFI; Medtech), MTF at distance and intermediate was unaffected by tilt up to ±2.5° or decentration up to ±0.5 mm with 3 mm and 4.5 mm pupils [89]. Ben Yaish et al. reported similar EDOF resilience, clear near–intermediate–distance vision, and astigmatism correction up to 1.00 D when decentration was 0.25 mm [90]. Breyer et al. confirmed that EDOF IOLs are more stable under decentration than the AcrySof ReSTOR SA60D3 (Alcon) bifocal IOL [8]. Xu et al. reported HOAs, coma, PSF, and glare perception were better in the monofocal IOLs and EDOF IOLs than those in the ZMB00 IOL when decentration was more than 0.25 mm [91].

## 8. Large Angle Kappa

The optical center of presbyopia-correcting IOLs should ideally align with the visual axis to ensure optimal performance. However, most IOLs are designed with symmetric, self-centering haptics that naturally position the lens within the capsular bag. This often results in temporal decentration relative to both the visual axis and the pupil center as observed commonly with angle kappa [18]. Misalignment among the visual axis, pupil center, and capsular bag center may cause light to strike the diffractive zones at unintended angles, leading to increased light scatter and visual disturbances such as halos and glare. Angle kappa is defined as the angular distance between the visual axis and the pupil center. Meng et al. reported an average angle kappa of approximately 0.3  ±  0.18 mm in Chinese population [92]. Wallerstein et al. reported angle kappa in 26,470 consecutive eyes that underwent immediate sequential bilateral cataract or refractive lens exchange with multifocal IOLs was 0.64 ± 0.27 mm in western population [93]. Because angle kappa is practically impossible to measure in the clinical setting, chord μ is frequently used to replace angle kappa. chord μ is not an angle but a two-dimensional Cartesian distance between the line of sight and the subject-fixated, coaxially sighted, corneal light reflex axis. The magnitude of chord μ roughly correlates with the Cartesian representation (mm) of angle kappa [18,94]. While earlier studies suggested that a large angle kappa (typically >0.5 mm) may contribute to reduced visual quality and a higher incidence of postoperative photic phenomena, recent evidence challenges its clinical relevance [93,95,96,97,98].

An excellent review on the significance of angle kappa in refractive surgery, including multifocal IOLs, was published by Kohnen et al. [18]. Numerous prospective and retrospective studies, including large-scale analyses, have found no significant association between preoperative angle kappa and postoperative outcomes such as visual acuity, refractive accuracy, or patient satisfaction [18,93,98]. While certain studies have linked larger angle kappa to increased photic phenomena—including halos, glare, and starbursts—others found no significant correlation [18,93]. Ang et al. reported that visual outcomes following implantation of Synergy (Johnson & Johnson Vision), PanOptix (Alcon), and FineVision (BVI Medical) remained favorable even in eyes with an angle kappa greater than 0.5 mm [98]. Liu et al. analyzed data from 1368 patients implanted with Tecnis Symfony (ZXTx, Johnson & Johnson Vision), Tecnis Multifocal 3.25 (Johnson & Johnson Vision), ReSTOR 2.5 ActiveFocus (Alcon), and Lentis MPlus (Oculentis B.V.), and found that angle kappa was not a predictive factor for patient-reported satisfaction [99]. These inconsistencies may be attributed to differences in pupil size, IOL design, measurement techniques, and study methodologies. Overall, there is still debate whether angle kappa alone is a reliable predictor of multifocal IOL candidacy or postoperative visual outcomes [93]. Its impact may vary depending on individual ocular anatomy, biometric parameters, and IOL design. Presbyopia-correcting IOLs are not an absolute contraindication for patients with a large angle kappa; however, thorough preoperative counseling is essential to ensure patients understand the potential risk of postoperative visual disturbances.

## 9. Choice of Presbyopia Correction Technology

Careful selection of an IOL that thoroughly reflects a patient’s lifestyle and visual demands is essential [5]. The surgeon should determine the patient’s priority working distances and explicitly ask whether those tasks must be performed without spectacles. Many patients require clear vision at multiple distances rather than a single focal point—for example, reading documents at 30 cm while typing keyboard viewing a monitor at 50 cm, driving at night while simultaneously seeing road signs, instrument panels, and an in-car navigation display, playing golf while tracking ball flight, putting and recording scores, or reading music note while observing a conductor across the stage. Patients who recall effortless near and intermediate vision in their twenties or thirties may expect presbyopia-correcting IOLs to fully restore that performance, giving rise to unrealistic expectations. The cataract surgeon must integrate these expectations with the patient’s ocular status, objective clinical findings, and the realistic benefits and limitations of available IOL technologies, then recommend the option that best balances probable visual outcomes with the patient’s priorities. The ultimate decision belongs to the informed patient after a comprehensive discussion, although many patients arrive at clinic trusting the surgeon to propose the optimal lens for their eyes and activities.

EDOF IOLs typically provide excellent distance and intermediate visual acuity but often fall short for near vision, with some studies showing acceptable near function for particular designs while overall evidence remains inconsistent [26]. Pure EDOF optics extend depth of focus through controlled aberrations or other optical manipulations but rarely achieve more than about 1 diopter of effective extension and thus cannot fully replace a dedicated near add, whereas multifocal IOLs produce simultaneous far, intermediate, and near images and rely on neuroadaptation to suppress unwanted focal images—failure of which results in dysphotopsias such as halos [8,100]. Hybrid or blended strategies that combine multifocal and EDOF concepts have been developed to leverage complementary strengths and mitigate limitations, and because EDOF lenses behave more like monofocals with modest near improvement, patients who require excellent unaided near vision are better served with a multifocal (refractive or diffractive) IOL.

Predicting how well patients will tolerate dysphotopic phenomena before surgery is very difficult. Simulators that reproduce postoperative experiences such as halos, blurring, and starbursts (positive dysphotopsias) and shadows or dimming (negative dysphotopsias) can be useful for patient counseling, but their predictive accuracy is limited, and their results should not be considered fully reliable [101,102]. In general, diffractive multifocal IOLs were more effective in improving unaided whole range of vision, but were associated with a higher rate of photic phenomena compared to EDOF IOL [10,13]. Therefore, patients who choose a diffractive multifocal IOL because of the importance of near tasks must receive thorough counseling about dysphotopsia and demonstrate clear understanding of its potential impact.

Mini-monovision and mix-and-match approaches using a low-add diffractive lens in one eye are commonly used to compensate EDOF’s near-vision shortfall. These strategies can increase spectacle independence but carry tradeoffs including reduced binocular distance acuity and the potential for additional halos or visual discomfort from induced low myopia in the fellow eye. Blended-vision regimens combining EDOF IOL with multifocal IOL are promising alternatives and can represent a viable option instead of trifocal implantation for patients prioritizing spectacle freedom. However this mix and match approach may increase the risk of dysphotopsia risk compared to monovision using EDOF IOLs [103]. Xiong et al. compared two IOL strategies: EDOF group receiving bilateral Symfony lenses, and a mix-and-match group combining Symfony with the Tecnis ZMB00 bifocal IOL. The mix-and-match approach yielded superior near vision outcomes; however, it was associated with a higher incidence of dysphotopsia, reported in 25% of patients compared to 5% in the EDOF group [103].

Neuroadaptation is a key determinant of subjective satisfaction with both multifocal and EDOF optics. Adaptation is time-consuming, varies between individuals, and may not fully compensate for a sudden increase in optical aberrations; therefore, pure EDOF lenses that rely on significant aberration to boost near vision may be poorly tolerated by some patients. Available data suggest that EDOF designs tend to produce less intense and smaller halos than multifocal lenses in some studies, but evidence remains limited. Rosa et al. found that multifocal IOL implantation produced a transient increase in cortical activity within networks subserving visual attention, procedural learning, effortful cognitive control, and goal-directed behavior in the early postoperative period, with these activations returning to baseline by six months [104]. Zhang et al. used functional MRI to compare the neuroadaptation after monofocal or multifocal IOL implantation. They found visual cortical function in the multifocal IOL group decreased at 1 week postoperatively and recovered to baseline at 3 months and then improved at 6 months, while visual cortical function increased at 1 week after surgery in the monofocal IOL group and decreased to baseline at 3 and 6 months [105].

If dissatisfied patients report visual discomfort soon after surgery but postoperative evaluation reveals no abnormalities in refraction, IOL centration, astigmatism, ocular surface, or macular condition, providing psychological support and allowing approximately six months for adaptation gives patients time to adjust to the novel visual experience produced by presbyopia-correcting IOLs. Proper preoperative counseling and realistic expectations are key to favorable outcomes. Patients generally confirm good vision and comfort [106].

## 10. Personality and Visual Outcome After Multifocal IOL Implantation

Personality exerts a pivotal influence on an individual’s cognitive appraisal of situational contexts, shaping whether experiences are interpreted positively or negatively. Patients exhibiting perfectionist tendencies—particularly those with exacting visual expectations—are more prone to dissatisfaction following multifocal IOL implantation [107]. Previous studies have reported that certain patient personality traits are associated with increased postoperative discomfort following multifocal IOL implantation.

Mester et al. studied visual satisfaction in over 130 patients at 6 month after multifocal IOL implantation (Tecnis ZM900, Tecnis ZMA00, ReZoom, ReSTOR+4 and ReSTOR+3, or AcriLISA). They found the personality characteristics of compulsive checking, orderliness, competence, and dutifulness were statistically significantly correlated to subjective disturbance by glare and halos [20]. Rudalevicius et al. compared visual satisfaction in 85 patients after implanting four types of multifocal IOLs (Rayner M-flex 630F, TECNIS ZMB00, AcrySofReSTOR or AT.LISAtri 839 MP), respectively [108]. Individuals characterized by a prevailing neurotic personality type demonstrated significantly less favorable visual satisfaction responses relative to patients with other dominant personality profiles. Pinheiro et al. studied the possible correlation between patients’ personality traits and subjective perception of quality of vision, after multifocal IOL implantation [109]. The study included 20 patients, evenly divided between those implanted with EDOF IOL (AcrySof^®^ IQ Vivity) and those with a trifocal IOL (AcrySof^®^ IQ PanOptix). At six months postoperatively, individuals with lower levels of conscientiousness and extraversion exhibited a significantly higher incidence of visual disturbances, including blurred vision, diplopia, and difficulty with focusing. Furthermore, patients with elevated neuroticism scores demonstrated increased difficulty in maintaining visual focus.

While it is impractical to perform formal personality assessments for every patient preoperatively, clinicians should use the brief encounter to attend closely to the patient’s stated needs, tone, attitude, and relevant medical history; when signs suggest perfectionistic tendencies, unrealistic surgical expectations, or traits consistent with high neuroticism, a more in-depth shared decision-making process and extended counseling about IOL options and likely outcomes are warranted.

Another critical aspect that must not be overlooked is that mental disorders such as anxiety and depression are common in older adults undergoing cataract surgery [110]. Studies in China reported anxiety and depression rates of 18% among cataract patients, compared with 7.0% and 5.2% in healthy controls. In Russia, anxiety prevalence was roughly 20% and depression rates among cataract patients were over 25% [111,112]. Furthermore, there are some studies suggesting that IOL with blue light blocking technology can increase the depression [113,114]. Therefore, blue-light-blocking multifocal IOLs or EDOF IOLs should be used with caution in patients with diagnosed depression because attenuation of short-wavelength light may worsen mood symptoms and potentially aggravate underlying mental illness. Although no comparative studies have been published to date, patients with depressive disorders may be at increased risk of dissatisfaction after implantation of multifocal or EDOF intraocular lenses.

An important fact is that many antidepressants used in clinical practice can cause pupil dilation. Selective serotonin reuptake inhibitors can cause pupil dilation by increasing serotonin levels in the brain, which influences the autonomic nervous system, while tricyclic antidepressants, due to their anticholinergic properties, may also lead to pupil dilation by inhibiting the iris sphincter muscle responsible for constricting the pupil [115,116,117]. Schmitt et al. reported the mesopic pupil size increased about 1.0 mm in selective serotonin reuptake inhibitors treatment group compared to placebo group [117]. These pupil change may affect the patient vision, especially after implantation of pupil size dependent presbyopia-correcting IOLs.

## 11. Postoperative Management

Previous studies have reported on the underlying causes of patient discomfort following the implantation of presbyopia-correcting IOLs, as well as the outcomes of therapeutic interventions aimed at addressing these issues. Wanten et al. analyzed 202 patients who underwent EDOF IOL implantation and found the major etiologies for dissatisfaction were residual ametropia (51.8%), dry eye disease (26.5%), and posterior capsular opacification (12.0%) [118] de Vries et al. included 76 eyes from 49 patients who complained discomfort after multifocal IOL implantation [11]. The implanted IOLs were ReSTOR (n = 73), Tecnis ZMA00 (n = 2), and ReZoom (n = 1). Blurred vision occurred in 72 eyes (94.7%), photic phenomena in 29 eyes (38.2%), and both symptoms in 25 eyes (32.9%). They found the leading causes of discomfort were residual ametropia or astigmatism, posterior capsule opacification, and large pupil. Conservative treatment failed to relieve the symptom in 3 cases (4.0%) and IOL exchange was required [11]. Woodward et al. analyzed 32 patients (43 eyes) with unwanted visual symptoms after multifocal IOL implantation: 28 eyes (65%) with AcrySof ReSTOR and 15 eyes (35%) with ReZoom. They found blurred vision in 30 patients (41 eyes), photic phenomena in 15 patients (18 eyes), and both in 13 patients (16 eyes). Blurred vision was caused by ametropia in 12 eyes (29%), dry eye in 6 eyes (15%), and PCO in 22 eyes (54%). Photic phenomena were caused by IOL decentration in 2 eyes (12%), retained lens fragment in 1 eye (6%), PCO in 12 eyes (66%), and dry eye 1 eye (2%). Conservative treatment failed in 3 eyes (7%), which required IOL exchange [17]. Gibbons et al. studied 74 eyes (49 patients) with dissatisfaction after the implantation of presbyopia-correcting intraocular lenses (IOLs). The different model lenses included in the study were ReSTOR (n = 44, SN6AD1 and SN6AD3), Crystalens (n = 17), Tecnis ZMA00 (n = 9), ReZoom (n = 3), and Array lens (n = 1). The most common complaint was blurry/foggy distance and near vision (68%). Primary causes were residual refractive error (57%) and dry eye (35%). Treatments included spectacles/contact lenses (46%), dry-eye therapy (24%), corneal laser (8%), and IOL exchange (7%). Although 45% of patients had complete symptom resolution, 23% showed partial improvement, and 32% remained completely dissatisfied [19].

Careful evaluation of IOL position during follow-up is essential to rule out clinically significant decentration, tilt, or rotation. Notable posterior capsule opacification may worsen visual symptoms, so earlier YAG capsulotomy should be considered when appropriate. Inspection of the ocular surface for tear-film deficiency and initiation of targeted dry-eye therapy are needed. Astigmatism control is critical for optimal presbyopia-correcting IOLs outcomes. Any significant or unexpected postoperative astigmatism should be monitored until stable and treated with appropriate measures such as limbal-relaxing incisions or other corneal refractive procedures. It is recognized that multifocal IOLs impose greater demands on minimal refractive error for patients’ satisfaction compared with EDOF IOLs [49,50]. Although precise comparative data are lacking in the literature, it is likely that secondary corneal refractive procedures are more frequently required in patients implanted with multifocal IOLs than in those with EDOF IOLs. Acknowledging these differences is important in both preoperative counseling and postoperative management, as it allows clinicians to tailor interventions to the specific optical characteristics of the implanted IOLs and to the patient’s visual expectations.

Patients receiving presbyopia-correcting IOLs may require closer and longer outpatient follow-up than those with monofocal IOLs. Maloney et al. reported that during the first postoperative year, presbyopia-correcting IOL patients spent a mean 5:50 ± 3:35 h (hour:minute) in the clinic across 6.6 ± 2.9 visits, versus 3:38 ± 1:36 h over 4.9 ± 1.6 visits for monofocal patients [119]. Mean visit duration of presbyopia-correcting IOL patients was 8 min longer, and they underwent more procedures and diagnostic tests than standard monofocal IOL cataract surgery patients [119].

Patient dissatisfaction with neuroadaptation failure following multifocal IOL implantation can be managed by multifocal IOL exchange with a different multifocal IOL optical profile or monofocal IOLs [120,121,122,123]. However, because dissatisfaction arises from multiple causes, patients may continue to experience visual disturbances even after multifocal IOL exchange. Patients should be clearly informed that exchanging a multifocal IOL for a monofocal IOL to address dysphotopsia often reduces near vision, and they must understand and accept this potential trade-off before surgery.

## 12. Conclusions

Presbyopia-correcting IOLs constitute a pivotal advancement in modern cataract surgery, enabling the restoration of functional vision across multiple focal distances. Nonetheless, the attainment of optimal postoperative satisfaction remains a multifaceted endeavor, influenced by the complex interplay of optical limitations, preexisting ocular pathology, surgical precision, and individual patient characteristics. While multifocal IOLs effectively enhance near and intermediate visual acuity, they are frequently associated with photic phenomena—such as glare and halos—that may compromise overall visual quality and patient satisfaction. Conversely, EDOF IOLs offer a more seamless visual experience, albeit with potential compromises in near-vision performance.

This review emphasizes the critical importance of comprehensive preoperative assessment, particularly in the detection and management of ocular surface disease, corneal irregularities, and glaucomatous changes, all of which may adversely affect surgical outcomes. Additionally, factors such as pupil diameter and IOL centration are shown to exert significant influence on optical performance, underscoring the necessity for meticulous surgical technique and personalized lens selection. Beyond anatomical and optical considerations, psychosocial determinants—including patient expectations, occupational demands, and psychological disposition—play a decisive role in shaping subjective satisfaction.

Although ongoing innovations in IOL design continue to enhance optical functionality, no single modality universally fulfills the diverse needs of all patients. Therefore, optimizing postoperative satisfaction with presbyopia-correcting IOLs mandates a holistic, patient-centered approach that integrates evidence-based optical principles with individualized clinical judgment. Through such tailored strategies, ophthalmic surgeons can more effectively align surgical outcomes with patient goals, thereby improving both visual function and quality of life.

## Figures and Tables

**Table 1:** Various Multifocal IOLs with different technologies.

Model	Manufacturer	Technology	Pupil Dependent	Add Power Diopter
Mplus LS-313 MF30	Oculentis, Berlin, Germany	refractive	no	+3.00 D
M-flex	Rayner, Worthing, UK	refractive	no	+3.00 D
Tecnis ZM900	Johnson & Johnson, New Brunswick, US	Diffractive	yes	+3.00 D
Tecnis ZMB100	Johnson & Johnson, New Brunswick, US	Diffractive	yes	+2.75 D
Acriva Trinova	VSY-Biotechnology, Leinfelden-Echterdingen, Germany	diffractive	yes	+3.00 D/trifocal/+1.50 D
FineVision HP	BVI Medical, Waltham, US	diffractive	yes	+3.50 D/trifocal/+1.75 D
AT LISA tri 839 MP	Zeiss, Oberkochen, Germany	diffractive	yes	+3.33 D/trifocal/+1.66 D
AT Elena 841P	Zeiss, Oberkochen, Germany	diffractive	yes	+3.33 D/trifocal/+1.66 D
PanOptix TFNT00 IOL	Alcon, Fort Worth, US	Diffractive; enlightened technology	no	+3.25 D/trifocal/+2.17 D
Tecnis Synergy	Johnson & Johnson, New Brunswick, US	Hybrid: combines extended-depth-of-focus (EDOF) optics with multifocal diffractive elements to deliver a continuous range of vision from distance through intermediate to near	yes	Enhanced depth of field
Tecnis Odyssey	Johnson & Johnson. New Brunswick, US	Diffractive: a novel nonparabolic diffractive profile, with nonuniform distribution of smoother, more rounded echelettes	no	Not disclosed

**Table 2:** Various EDOF IOLs with different technologies.

Model	Manufacturer	Technology	Pupil Dependence	Add Power
Mini Well Ready	SIFI, Medtech, Aci S. Antonio, Italy	Refractive; Three optical zones: an outer monofocal zone, a 1.80 mm inner zone with positive spherical aberration for intermediate focus, and a 3.00 mm middle zone with negative spherical aberration to enhance near focus.	Yes	+3.00 D
Lentis Comfort	Teleon Surgical, Spankeren, Netherlands	Refractive; a rotationally asymmetric multifocal IOL with +1.50 D near addition	No	+1.50 D
Acunex Vario	Teleon Surgical, Spankeren, Netherlands	Refractive; a rotationally asymmetric multifocal IOL with +1.50 D near addition	unknown	+1.50 D
IC-8	AcuFocus, Irvine, US	Pinhole Effect; A 3.23 mm black annular mask made of polyvinylidene difluoride and carbon nanoparticles blocks defocused light, while a central 1.36 mm clear aperture permits paraxial rays, producing an EDOF effect.	No	+3.00 D
AT LARA 829 MP	Carl Zeiss Meditec, Jena, Germany	Diffractive; a continuous diffractive surface profile from intermediate to distance focal points	Yes	0.95 D, +1.90 D
Symfony ZXR00	Johnson & Johnson, New Brunswick, US	Diffractive; a biconvex, wavefront-optimized anterior aspheric surface (−0.27 μm) combined with a posterior achromatic diffractive pattern utilizing an echelette design for enhanced optical performance.	Yes	+1.75 D
FineVision Triumf	PhysIOL, Liège, Belgium	Diffractive; The design integrates two bifocal diffractive grating elements—one optimized for distance and near vision (+3.50 D add), and the other for distance and intermediate vision (+1.75 D add)—to enhance visual performance across multiple ranges	Yes	+1.75 D, +3.50 D
Lucidis	SAV-IOL SA, Neuchâtel, Switzerland	Refractive; The 1 mm central aspheric zone functions as an axicon, generating a Bessel beam that produces a continuous range of focal fields, enabling seamless vision from intermediate to near distances	Yes	+3.00 D
EDEN	SAV-IOL SA, Neuchâtel, Switzerland	Refractive/Diffractive; This lens features a hybrid refractive-diffractive design: a 1 mm central aspheric zone enhances near and intermediate vision, encircled by a 3.5 mm diffractive zone for near and distance focus, and a 6 mm outer refractive zone dedicated to distance vision.	Yes	+3.00 D
Harmonis	SAV-IOL SA, Neuchâtel, Switzerland	Refractive/Diffractive; This lens features a hybrid refractive-diffractive design: a 1.5 mm central aspheric zone enhances near and intermediate vision, encircled by a 3.7 mm diffractive zone for near and distance focus, and a 6 mm outer refractive zone dedicated to distance vision.	possible	+2.50 to +3.50 D
EyHance ICB00	Johnson & Johnson, New Brunswick, US	Refractive monofocal EDOF; incorporates a small central plateau that induces a localized refractive power change, while its anterior surface features an aberration-correcting curvature with negative primary spherical aberration.	Yes	+1.50 D
AE2UV/ZOE	Eyebright/Ophthalmo Pro GmbH, St. Ingbert, Germany	Refractive monofocal EDOF; A high-order aspheric surface enhances intermediate vision by introducing elevated spherical aberration at the IOL center, which progressively diminishes toward the periphery.	possible	+0.75 to 1.00 D
Synthesis PLUS	Cutting Edge, Montpellier, France	Refractive monofocal EDOF; a combination of primary and secondary SAs of opposite signs promoting an increase of the depth of field	possible	+1.50 D
Vivity DFT015	Alcon, Fort Worth, US	Refractive monofocal EDOF; nondiffractive X-WAVE technology which stretches and shifts light	No	+1.50 D
LuxSmart	Bausch & Lomb, Vaughan, Canada	Refractive; a combination of 4th and 6th orders of SA of opposite sign	possible	+1.75 D
RayOne EMV	Rayner, Worthing, UK	Refractive monovision; an increased positive SA to enhance the depth of focus, while the lens outer periphery behaves aberration-neutral	No	Up to 2.25 D EDOF with 1.00 D offset
PureSee	Johnson & Johnson, New Brunswick, US	Refractive; a combination of 4th and 6th orders of SA of opposite sign	possible	+1.75 diopters at the spectacle plane (2.2 D at the IOL plane)
Galaxy	Rayner, Worthing, UK	Spiral optic design increased depth of focus	less pupil dependency	Approximately 4 D range of 0.2 logMAR or better near vision

## Data Availability

No new data were created or analyzed in this study.
